# Comparison of all-cause mortality risk factors in a population-based cohort study

**DOI:** 10.1038/s41598-026-44015-4

**Published:** 2026-03-28

**Authors:** Carlos Lederman, Joany Mariño Coronado, Nágila Raquel Teixeira Damasceno, Sabine Schipf, Alfredo José Mansur, Nele Friedrich, Matthias Nauck, Henry Völzke, Marcus Dörr, Davide Di Vece, Christian Templin, Martin Bahls, Till Ittermann, Marcello Ricardo Paulista Markus

**Affiliations:** 1 Instituto do Coração (InCor) do Hospital das Clínicas da Faculdade de Medicina da Universidade de São Paulo (HCFMUSP), São Paulo, Brazil; 2 Department of Internal Medicine B, University Medicine Greifswald, Greifswald, Germany; 3 German Centre for Cardiovascular Research (DZHK), Partner Site Greifswald, Greifswald, Germany; 4 Nutrition Department, School of Public Health, University of São Paulo, São Paulo, Brazil; 5 Postgraduate Program in Cardiology, Heart Institute (InCor), Faculty of Medicine, University of São Paulo (FMUSP), São Paulo, Brazil; 6 Department of Study of Health in Pomerania/Clinical-Epidemiological Research, Institute for Community Medicine, University Medicine Greifswald, Greifswald, Germany; 7 Institute of Clinical Chemistry and Laboratory Medicine, University Medicine Greifswald, Greifswald, Germany; 8 First Clinic of Internal Medicine, Department of Internal Medicine, University of Genoa, Genoa, Italy

**Keywords:** lifestyle, metabolic factors, nomogram, prospective cohort study, sex differences, social determinants, Cardiology, Diseases, Endocrinology, Health care, Medical research, Risk factors

## Abstract

All-cause mortality is a population health indicator of the combined impact of biological, behavioral, social, and healthcare-related factors. We used data from 3,803 participants (1,947 women, 51.2%; aged 20 to 81 years) of the population-based Study of Health in Pomerania (SHIP-START-0, 1997–2001), with a median follow-up duration of 20.2 years. Sex-stratified cox proportional hazard models were used to estimate associations between socioeconomic, lifestyle, anthropometric, and cardiovascular risk factors with all-cause mortality. During the 70,982 person-years, 1,029 deaths (641 men and 388 women) were determined as all-cause mortality. In men, type 2 diabetes (hazard ratio [HR] = 1.83 [95% confidence interval {CI}: 1.48 to 2.25; *p* < 0.001]), living without a partner (HR = 1.78 [95% CI: 1.41 to 2.24; *p* < 0.001]), being a current smoker (HR = 1.76 [95% CI: 1.41 to 2.20; *p* < 0.001]), older age (HR per year = 1.10 [95% CI: 1.10 to 1.11; *p* < 0.001]) and elevated hs-CRP (HR per mmol/l = 1.07 [95% CI: 1.03 to 1.11; *p* < 0.001]) where significantly associated with increased all-cause mortality. In women, just type 2 diabetes (HR = 1.70 [95% CI: 1.28 to 2.15; *p* < 0.001]) and elevated hs-CRP (HR per mmol/l = 1.07 [95% CI: 1.03 to 1.12; *p* < 0.001]) where significantly associated with increased all-cause mortality. Type 2 diabetes and inflammation were linked to higher all-cause mortality in both sexes, whereas being without a partner, current smoking, and older age were significant risk factors specifically for men.

## Introduction

All-cause mortality, defined as death resulting from any cause within a given population over a specified period of time, is considered the best objective outcome without bias^1,2^. While factors associated to the cause of death may be difficult to establish, all-cause mortality provides an objective measure of general health outcomes that represents the sum consequences of multiple risk factors, comorbidities and health interventions. Contrary to cause-specific mortality, all-cause mortality avoids the risk of misclassification, which results in a clearer representation of the burden of diseases and the effects of lifestyle, socioeconomic, environmental and healthcare factors on a population^3,4^.

Large population-based cohort studies are commonly used to show trends and determinants of all‐cause mortality along regions, time periods and demographic groups. Previous studies^3,4^ showed that modifiable risk factors such as physical activity, dietary patterns, social isolation and chronic diseases may affect mortality rates. A recent analysis^3^ from an extensive cohort data in the United States, with 179 million deaths over 1900 to 2000, showed major disparities in all-cause mortality trends by birth cohort, state, and sex, highlighting the complexity of underlying factors and the need for targeted public health interventions. The broad measurement of all‐cause mortality in well‐conducted cohorts yields critical insights for public health, by identifying the most impactful health priorities and informing interventions to reduce death rates in diverse populations^3,4^.

The Study of Health in Pomerania (SHIP)^5–7^ provides a broad context for studying mortality due to its representative sample of adults and its longitudinal design. SHIP is an established prospective health study in north-eastern Germany that enrolled a representative sample of adults. This comprehensive data set has been used to study chronic diseases, risk factors and health outcomes specific to the West Pomeranian population in Germany, confirming for example an unusually high accumulation of common risk factors in this region^8^. In the present study, we used the SHIP cohort to analyze how demographic, clinical and lifestyle factors relate to the risk of all-cause mortality among its participants.

## Methods

### Study population

The analysis is based on data from the baseline examination SHIP-START-0 conducted between 1997 and 2001. The study design has been formerly described^5–7,9^. In summary, a sample was randomly selected adults aged 20 to 79 years via a multistage process in the cities of Greifswald, Stralsund and Anklam and 29 communities in the surrounding region which is part of West Pomerania and is the most north-easterly region of Germany, adjacent to the Baltic Sea in the north and to the Polish border in the east. The sample selection was done in two steps. First, of the three districts in the region, the three cities (17,076 to 65,977 inhabitants) and the 12 towns (1,516 to 3,044 inhabitants) were selected, and of the small towns (less than 1,500 inhabitants), 17 out of 97 were drawn at random. Second, from each of the selected communities, subjects were drawn at random from official inhabitant data files, proportional to the population size of each community and stratified by age and gender. The addresses include those living institutionalized too. Since there was just a proportion of 1.6% Non-German citizens in the population only individuals with German citizenship and main residency in the study area were included. The total population within the recruitment area consisted of 212,157 residents. A total of 7,008 participants were sampled with 292 individuals of each sex in each of the twelve 5-year age strata. The net sample (excluding those who have migrated or deceased) consisted of 6,265 eligible individuals. A maximum of three written invitations were sent to the selected persons. If there was no response, letters were then followed by a phone call or by home visits when phone contact was not feasible. The final SHIP-START-0 population consisted of 4,308 individuals, of whom 2,192 were women (50.9%), representing a response rate of 68.8%^9^. In the presented study, participants were excluded if they had missing values data for any of the variables being analyzed (*n* = 505). The final analytical sample consisted of 3,803 individuals, aged from 20 to 81 years, including 1,947 women who made up 51.2% of the group at the initial examination.

All participants provided written informed consent prior to the study. The study received approval from the ethics committee of the University of Greifswald^6^ and adheres to the Declaration of Helsinki.

### Interview, medical and laboratory examination

Trained and certificated medical staff collected information on age, sex, socioeconomic determinants (including education, household equivalent income in euros and living with a partner or alone), smoking habits, and medical history during a standardized interview^9^.

Education was classified based on years of schooling into low (less than ten years), middle (ten years), and high (more than ten years) categories. Smoking status was categorized as never smoker, former smoker, and current smoker, the latter being those who at the time of the interview consumed at least one cigarette per day. Previous month alcohol consumption was measured by drinking behavior. The daily intake of pure alcohol (g/day) was calculated by multiplying the number of days on which alcohol was consumed by the average number of alcoholic drinks consumed on those days and then dividing by 30. The result was then adjusted using standard alcohol concentrations of 4.8% for beer, 11.0% for wine, and 33.0% for spirits^8^. Individuals were classified as inactive if they did not engage in recreational exercise for a minimum of one hour per week during summer or/and winter^9,10^.

An extensive standardized medical examination was performed on all participants, including the collection of blood samples. Anthropometric measurements consisted of height and weight, as per the guidelines of the World Health Organization^9,11^. Body weight and height were recorded to the nearest 0.1 kg and 0.5 cm, respectively, using calibrated weighing scales and stadiometers, with participants wearing light clothing and no shoes. The body mass index (BMI) was determined by dividing weight in kilograms by the square of height in meters. Waist circumference was measured to the nearest 0.1 cm, using an inelastic tape positioned midway between the lower rib margin and the iliac crest, in a horizontal plane, with the participant standing comfortably with their weight evenly distributed on both feet^9,12^. The examiner measured the hip circumference, in centimeters, by palpating the most lateral point of the greater trochanter and the iliac crest and then taking the measurement midway between these two points. Waist-to-hip and waist-to-height ratios were calculated by dividing the waist circumference by the hip circumference and body height, respectively. Both ratios were then multiplied by 100 to display the results as percentages.

Blood pressure and heart rate were measured three times on the right arm of seated participants, employing an oscillometric digital blood pressure monitor (HEM-705CP, Omron Corporation, Tokyo, Japan) following a resting period of at least five minutes, with a three-minute interval between each reading. Systolic and diastolic blood pressure means were calculated from the second and third measurements and were employed for the current analysis. Hypertension was defined as systolic blood pressure being 140 mm Hg or higher and/or diastolic blood pressure being 90 mm Hg or higher and/or current self-reported use of any anti-hypertensive medication^9^.

While they were seated, all study participants had non-fasting venous blood samples collected between 07:00 a.m. and 04:00 p.m^9,13^. Serum aliquots were stored at a temperature of minus 80 Celsius degrees. All assays were performed according to the manufacturers’ recommendations guidelines by experienced technical staff^9,14^. Serum glucose concentrations were determined enzymatically using reagents from Roche Diagnostics (Hitachi 717, Roche Diagnostics, Mannheim, Germany)^9,15^. Glycated hemoglobin was determined by high-performance liquid chromatography (Diamat, Bio-Rad Laboratories, Munich, Germany)^16^. Type 2 diabetes was diagnosed based on self-reported information and/or a glycated hemoglobin ≥ level of 6.5% or higher, and/or a random glucose ≥ level of 11.1 mmol/l or higher, and/or current self-reported use of any hypoglycemic medication classified by the ATC code A10. Total serum cholesterol, low-density lipoprotein cholesterol (LDL-C), and high-density lipoprotein cholesterol (HDL-C) were measured photometrically (Hitachi 704, Roche Diagnostics, Mannheim, Germany)^16,17^. Triglycerides concentrations were determined enzymatically using reagents from Roche Diagnostics (Hitachi 717, Roche Diagnostics, Mannheim, Germany)^9,17^.

Total cholesterol / HDL-C and triglycerides / HDL-C ratios were calculated as the total cholesterol and triglycerides, respectively, divided by HDL-C. Lipoprotein(a) (Lp[a]) concentrations were measured by an immunoluminometric assay using two polyclonal antibodies directed against apolipoprotein(a) on a Magic Lite Analyzer II (Ciba Corning, Fernwald, Germany)^9,18^. Hypercholesterolemia was diagnosed based on serum cholesterol ≥ 6.2 mmol/l and/or LDL-C ≥ 4.1 mmol/l mmol/l and/or total cholesterol/HDL-cholesterol ratio ≥ 5.0 and /or self-reported use of any lipid-lowering medication classified by the ATC code C10^9,19^. High-sensitivity C-reactive protein (hs-CRP) concentrations were determined immunologically on a Behring Nephelometer II with commercially available reagents (Dade Behring, Eschborn, Germany)^15,16^. Serum creatinine concentration was assessed using a modified kinetic Jaffé method (Hitachi 717, Roche Diagnostics, Mannheim, Germany)^17^. The estimated glomerular filtration rate (eGFR) was determined according to the Chronic Kidney Disease – Epidemiology Collaboration (CKD-EPI) equation^20^ and expressed in mL/min/1.73 m^2^: eGFR = 141 × min (serum creatinine/κ)^α^ × max (serum creatinine/κ) − 1.209 × 0.993^age^× 1.018 (if women) × 1.159 (if black) where K is 0.7 for women and 0.9 for men, α is -0.329 for women and − 0.411 for men, min indicates the minimum of serum creatinine/κ or 1, and max indicates the maximum of serum creatinine / κ or 1. Since all study participants were of European Caucasian descent, the race variable was omitted from the equation.

Transabdominal ultrasound of the liver was performed by examiners using a transportable B-mode ultrasound device. Hepatic steatosis was defined as a hyperechogenic liver pattern in comparison to the renal cortex^6^. History of non-fatal myocardial infarction or stroke was self-reported.

### Information on vital status

Information on vital status was collected at regular intervals from time of enrolment into the study through March 31, 2019^9^. Two certified internists independently coded the underlying cause of death according to the International Classification of Diseases version 10 (ICD-10) using death certificates obtained from the local health authorities at the place of death^9^. In the event of a disagreement, a joint reading was undertaken.

Participants were censored at death or loss to follow-up. The number of months between baseline examination and censoring was used as the duration of follow-up^9^.

### Statistical analysis

Descriptive data was reported as median (25th and 75th percentile) for continuous variables and as absolute numbers and percentages for categorical variables stratified by sex and vital status.

Associations of socioeconomic factors (education, household equivalent income, living with / without a partner), life style factors (smoking status, alcohol consumption, sedentarism), anthropometric factors (body height and weight, BMI, BMI status, WC, hip circumference, waist-to-hip and waist-to-height ratios), and cardiovascular risk factors (systolic and diastolic blood pressure, random glucose, glycated hemoglobin, total cholesterol, LDL-C, HDL-C, TG, lipoprotein (a), eGFR, hs-CRP, prevalent hypertension, type 2 diabetes, hypercholesterolemia, eGFR < 60 mL/min/1.73 m^2^, hepatic steatosis, non-fatal myocardial infarction and stroke, and use of antihypertensive, glucose lowering and lipid-lowering medication) with all-cause mortality were analyzed by Cox proportional hazard models adjusted for the baseline age and stratified by sex. To make the regression models comparable, the continuous variables were standardized. One standard deviation (SD) corresponded to 16.4 years for age, 491 euros for household equivalent income, 18.6 g/day for alcohol consumption, 9.43 cm for body height, 15.2 kg for body weight, 4.74 kg/m^2^ for BMI, 13.8 cm for WC, 9.78 cm for hip circumference, 8.87% for waist-to-hip ratio, 8.11% for waist-to-height ratios, 20.9 mm Hg for systolic blood pressure, 11.2 mm Hg for diastolic blood pressure, 1.67 mmol/L for random glucose, 0.90% for glycated hemoglobin, 1.10 mmol/L for total cholesterol, 0.92 mmol/L for LDL-C, 0.40 mmol/L for HDL-C, 1.86 for total cholesterol / HDL-C ratio, 1.18 mmol/L for TG, 2.14 for triglycerides / HDL-C ratio, 286 mg/L for lipoprotein (a), 2.50 mg/L for hs-CRP, and 19.1 mL/min/1.73 m^2^ for eGFR. The following continuous variables were additionally adjusted for the use of antihypertensive medication (systolic and diastolic blood pressure), glucose-lowering medication (random glucose and glycated hemoglobin) and lipid-lowering medication (total cholesterol, LDL-C, HDL-C, total cholesterol / HDL-C ratio, triglycerides, and triglycerides / HDL-C ratio).

We used a backward (*p* ≥ 0.001 for removal) elimination procedure to identify the sex-specific markers most strongly associated with all-cause mortality and calculated Harrel’s C from these models as a measure of discrimination.

A two-sided p-value *p* < 0.05 was considered as statistically significant in all analyses. All calculations were performed using Stata 18.5 (Stata Corporation, College Station, TX, USA).

Finally, we developed sex-specific nomograms based on the multivariable Cox proportional hazards prediction models. Using the nomogram function from the rms package in R, we assigned point values to each predictor variable according to its relative contribution to the model. The total points for an individual correspond to the estimated probability of death, which we calculated for 5-, 10-, and 20-years.

## Results

A total of 3,803 participants were followed with a median duration of 20.2 years (interquartile range: 18.6 to 21.9). During the 70,982 person- years, 1,029 deaths (641 men and 388 women) were determined as all-cause mortality.

Table 1 shows the baseline and follow-up characteristics of the study population stratified by sex and vital status. When compared with living men, deceased men were older, with lower education and household income, more usually living with a partner, former smokers, sedentary, had lower alcohol consumption, height and weight, but with a higher BMI, WC, hip circumference, waist-to-hip and waist-to-height ratios with a consequently more prevalence of obesity. Deceased men also showed lower concentrations of HDL-C, but higher systolic blood pressure, random glucose, glycated hemoglobin, total cholesterol/ HDL-C ratio, triglycerides, lipoprotein (a), and hs-CRP levels. They were also more likely to have a history of hypertension, type 2 diabetes, hypercholesterolemia, lower renal function and a greater use of antihypertensive, glucose-lowering, and lipid-lowering medications. On the other hand, total cholesterol and LDL-C levels were similar between the living and deceased groups. Noteworthy, the group of deceased men had also higher prevalence of hepatic steatosis, and previous non-fatal myocardial infarction and stroke.

**Table 1:** Characteristics of the study population stratified by sex and vital status (*n* = 3,803).

	All-cause mortality			
	Men		Women	
	Alive	Dead	Alive	Dead
N (%)	1,215 (65.5)	641 (34.5)	1,559 (80.1)	388 (19.9)
Age (years)	42 (32; 54)	68 (59; 74)	43 (33; 55)	69 (62; 75)
Education (%)				
Low	28.0	69.0	27.0	76.8
Middle	51.9	19.5	55.2	18.0
High	20.2	11.5	17.8	5.20
Household equivalent income (euros)	959 (627; 1342)	949 (678; 1175)	859 (600; 1175)	949 (703; 1097)
Living with partner (%)	79.3	82.4	75.9	49.9
Smoking status (%)				
Lifetime non-smokers	23.9	16.2	46.0	63.4
Former smokers	38.6	56.6	23.5	21.9
Current smokers	37.5	27.1	30.5	14.7
Alcohol consumption (g/day)	13.4 (4.6; 28.5)	6.3 (0.9; 18.0)	3.9 (1.3; 8.6)	0.7 (0.0; 4.0)
Sedentarism (%)	52.2	70.8	52.3	72.2
Body height (cm)	177 (172; 181)	172 (168; 177)	164 (159; 168)	159 (154; 163)
Body weight (kg)	85 (76; 93)	82 (75; 92)	68 (60; 79)	73 (64; 82)
BMI (kg/m^2^)	27.0 (24.6; 29.5)	28.1 (25.4; 30.6)	25.4 (22.5; 29.3)	28.8 (25.4; 32.6)
BMI status (%)				
BMI < 25	29.5	21.2	47.3	21.9
Overweight	49.0	49.5	30.3	37.6
Obesity	21.6	29.3	22.4	40.5
Waist circumference (cm)	93 (85; 100)	99 (92; 106)	79 (72; 90)	89 (82; 100)
Hip circumference (cm)	101 (97; 106)	104 (99; 108)	100 (94; 108)	108 (100; 115)
Waist-to-hip-ratio (%)	92 (87; 96)	95 (91; 99)	79 (75; 84)	83 (80; 87)
Waist-to-height-ratio (%)	53 (48; 57)	58 (54; 62)	48 (43; 55)	56 (51; 63)
Systolic blood pressure (mmHg)	138 (126; 148)	148 (134; 162)	123 (112; 137)	142 (130; 154)
Diastolic blood pressure (mmHg)	85 (78; 92)	85 (78; 94)	80 (73; 87)	82 (75; 89)
Hypertension (%)	53.3	79.7	34.2	71.4
Antihypertensive medication (%)	15.8	53.2	17.6	57.2
Random glucose (mmol/L)	5.4 (5.0; 5.8)	5.7 (5.1; 6.6)	5.1 (4.7; 5.5)	5.5 (5.0; 6.2)
Glycated hemoglobin (%)	5.2 (4.9; 5.6)	5.7 (5.2; 6.2)	5.1 (4.8; 5.5)	5.7 (5.2; 6.3)
Type 2 diabetes (%)	5.3	24.0	4.6	26.3
Glucose-lowering medication (%)	2.2	13.3	2.6	16.5
Total cholesterol (mmol/L)	5.3 (4.6; 6.0)	5.3 (4.6; 6.0)	5.2 (4.5; 6.0)	5.8 (5.1; 6.5)
LDL-cholesterol (mmol/L)	3.3 (2.8; 3.9)	3.3 (2.7; 3.9)	3.1 (2.5; 3.7)	3.6 (3.0; 4.1)
HDL-cholesterol (mmol/L)	1.13 (0.93; 1.35)	1.06 (0.88; 1.28)	1.41 (1.17; 1.67)	1.27 (1.02; 1.58)
Total cholesterol / high-density lipoprotein cholesterol ratio	4.7 (3.7; 5.9)	4.9 (3.9; 6.1)	3.7 (2.9; 4.6)	4.5 (3.6; 5.8)
Triglycerides (mmol/L)	1.7 (1.2; 2.6)	1.8 (1.3; 2.7)	1.3 (0.9; 1.9)	1.8 (1.3; 2.5)
Triglycerides / high-density lipoprotein cholesterol ratio	1.52 (0.91; 2.57)	1.70 (1.09; 2.90)	0.91 (0.59; 1.46)	1.35 (0.86; 2.37)
Lipoprotein(a) (mg/L)	89 (41; 248)	91 (44; 262)	94 (44; 272)	116 (49; 292)
Hypercholesterolemia (%)	50.2	64.7	34.3	63.7
Lipid-lowering medication (%)	5.1	18.9	4.8	14.7
High-sensitivity C-reactive protein (mg/L)	1.0 (0.5; 2.0)	2.0 (0.9; 4.2)	1.3 (0.6; 3.2)	2.0 (0.9; 4.6)
Estimated glomerular filtration rate (CKD-EPI*) (mL/min/1.73 m^2^)	107 (96; 116)	89 (75; 99)	105 (93; 116)	86 (72; 95)
Estimated glomerular filtration rate (CKD-EPI*) < 60 mL/min/1.73 m^2^ (%)	0.8	8.5	0.6	8.9
Hepatic steatosis (%)	32.8	46.9	17.7	39.7
Non-fatal myocardial infarction (%)	1.6	9.6	0.5	3.1
Non-fatal stroke (%)	0.7	5.2	0.3	3.1

Data are expressed as median, 25th, and 75th percentile for continuous data or as percentages for categorical data.

*CKD-EPI: Chronic Kidney Disease – Epidemiology Collaboration equation^20^.

When compared with living women, deceased women were older, had lower levels of education, but higher house income, and were more likely to live without a partner. They were also, more often former smokers, sedentary, had lower alcohol consumption and shorter height, but higher weight, BMI, WC, hip circumference, waist-to-hip and waist-to-height ratios, with a correspondently higher prevalence of obesity. Deceased women also showed lower concentrations of HDL-C, but higher systolic and diastolic blood pressure, random glucose, glycated hemoglobin, total cholesterol, LDL-C, total cholesterol/ HDL-C ratio, triglycerides, lipoprotein (a), and hs-CRP levels. They were also more likely to have a history of hypertension, type 2 diabetes, hypercholesterolemia, lower renal function and a greater use of antihypertensive, glucose-lowering, and lipid-lowering medications. Noteworthy, the deceased women group had also higher prevalence of hepatic steatosis, and previous non-fatal myocardial infarction and stroke.

### Associations of risk factors with all-cause mortality

In sex-stratified Cox proportional hazard models adjusted for the baseline age (not for age), we demonstrated significant associations of risk factors with all-cause mortality (Table 2). Men had a 89% (95% confidence interval: 67 to 115; *p* < 0.001) higher risk for all-cause mortality when compared to women. In men, age (+ 381% per one SD) was the strongest risk exposure for all-cause mortality followed by being a current smoker. Specifically, compared with lifetime no-smokers, the current smokers had a 149% higher chance of all-cause mortality. Other risk factors associated with all-cause mortality were use of glucose-lowering medication (+ 93%), living without a partner (+ 89%), previous history of type 2 diabetes (+ 85%), non-fatal stroke (+ 70%), lower education (+ 45%), and use of antihypertensive medication (+ 32%), higher values of hs-CRP (+ 27% per one SD), waist-to-hip (+ 23% per one SD) and waist-to-height (+ 19% per one SD) ratios, being sedentary (+ 19%), having higher WC (+ 16% per one SD), glycated hemoglobin (+ 16% per one SD), triglycerides (+ 14% per one SD), triglycerides / high-density lipoprotein cholesterol (+ 11% per one SD) and total cholesterol / high-density lipoprotein cholesterol (+ 8% per one SD) ratios, and random glucose (+ 9 per one SD). Contrary to that, higher household equivalent income (− 19% per one SD) and LDL-C (− 8% per one SD) were associated with lower all-cause mortality. On the other hand, alcohol consumption, body height and weight, BMI, BMI status, hip circumference, systolic and diastolic blood pressure, total cholesterol, HDL-C, Lp(a), eGFR, and previous history of hypertension, hypercholesterolemia, use of lipid-lowering medication, eGFR below 60 mL/min/1.73 m^2^, hepatic steatosis, and myocardial infarction were not associated with all-cause mortality in men.

**Table 2:** Adjusted* hazard ratios (HR) (95% confidence interval [CI]; p-value) of the associations of risk factors with all-cause mortality stratified by sex (*n* = 3,803).

Parameter	All-cause mortality HR (95% CI); *p*-value	
	Men (*n* = 1,856)	Women (*n* = 1,947)
Socioeconomic factors		
Education		
High	1	1
Middle	1.08 (0.81 to 1.44); *p* = 0.612	1.41 (0.85 to 2.31); *p* = 0.180
Low	1.45 (1.12 to 1.86); *p* = 0.004	1.78 (1.12 to 2.83); *p* = 0.015
Household equivalent income (SD)**	0.81 (0.73 to 0.89); *p* < 0.001	0.87 (0.76 to 1.01); *p* = 0.064
Living with a partner	1	1
Living without a partner	1.89 (1.54 to 2.32); *p* < 0.001	1.09 (0.87 to 1.35); *p* = 0.461
Life style factors		
Smoking status		
Lifetime non-smokers	1	1
Former smokers	1.19 (0.96 to 1.48); *p* = 0.121	1.11 (0.87 to 1.42); *p* = 0.411
Current smokers	2.49 (1.94 to 3.21); *p* < 0.001	1.62 (1.20 to 2.19); *p* = 0.002
Alcohol consumption (SD)	1.03 (0.97 to 1.08); *p* = 0.357	0.91 (0.64 to 1.30); *p* = 0.618
No sedentarism	1	1
Sedentarism	1.19 (1.00 to 1.42); *p* = 0.045	1.42 (1.14 to 1.78); *p* = 0.002
Anthropometric factors		
Body height (SD)	0.96 (0.85 to 1.08); *p* = 0.469	1.03 (0.87 to 1.21); *p* = 0.755
Body weight (SD)	1.02 (0.92 to 1.12); *p* = 0.733	1.23 (1.10 to 1.39); *p* < 0.001
Body mass index (SD)	1.04 (0.94 to 1.16); *p* = 0.418	1.18 (1.07 to 1.30); *p* = 0.001
Body mass index status		
Normal weight (< 25 kg/m^2^)	1	1
Overweight (25 to 29.9 kg/m^2^)	0.82 (0.67 to 1.00); *p* = 0.050	1.04 (0.79 to 1.36); *p* = 0.776
Obesity (≥ 30 kg/m^2^)	1.00 (0.81 to 1.25); *p* = 0.967	1.32 (1.01 to 1.72); *p* = 0.042
Waist circumference (SD)	1.16 (1.05 to 1.29); *p* = 0.004	1.29 (1.15 to 1.45); *p* < 0.001
Hip circumference (SD)	1.09 (0.98 to 1.21); *p* = 0.108	1.20 (1.09 to 1.31); *p* < 0.001
Waist-to-hip-ratio (SD)	1.23 (1.08 to 1.39); *p* = 0.002	1.21 (1.05 to 1.40); *p* = 0.009
Waist-to-height- ratio (SD)	1.19 (1.07 to 1.33); *p* = 0.001	1.24 (1.12 to 1.38); *p* < 0.001
Cardiovascular risk factors		
Age (SD)	4.81 (4.27 to 5.42); *p* < 0.001	7.14 (6.02 to 8.46); *p* < 0.001
Systolic blood pressure (SD)	1.06 (0.98 to 1.16); *p* = 0.156	1.04 (0.93 to 1.16); *p* = 0.485
Diastolic blood pressure (SD)	0.96 (0.89 to 1.04); *p* = 0.311	0.98 (0.88 to 1.09); *p* = 0.744
No hypertension	1	1
Hypertension	1.21 (0.99 to 1.48); *p* = 0.057	1.21 (0.97 to 1.53); *p* = 0.093
No use of antihypertensive medication	1	1
Antihypertensive medication	1.32 (1.11 to 1.56); *p* = 0.001	1.39 (1.12 to 1.72); *p* = 0.002
Random glucose (SD)	1.09 (1.02 to 1.17); *p* = 0.009	1.03 (0.95 to 1.12); *p* = 0.461
Glycated hemoglobin (SD)	1.16 (1.07 to 1.25); *p* < 0.001	1.14 (1.03 to 1.25); *p* = 0.007
No type 2 diabetes	1	1
Type 2 diabetes	1.85 (1.54 to 2.23); *p* < 0.001	1.62 (1.28 to 2.04); *p* < 0.001
No use of glucose-lowering medication	1	1
Glucose-lowering medication	1.93 (1.53 to 2.44); *p* < 0.001	1.75 (1.33 to 2.29); *p* < 0.001
Total cholesterol (SD)	0.95 (0.87 to 1.03); *p* = 0.216	0.98 (0.88 to 1.08); *p* = 0.646
Low-density lipoprotein cholesterol (SD)	0.92 (0.84 to 1.00); *p* = 0.048	1.01 (0.90 to 1.12); *p* = 0.918
High-density lipoprotein cholesterol (SD)	0.91 (0.82 to 1.00); *p* = 0.051	0.86 (0.77 to 0.95); *p* = 0.005
Total cholesterol / high-density lipoprotein cholesterol ratio (SD)	1.08 (1.01 to 1.15); *p* = 0.021	1.20 (1.08 to 1.35); *p* = 0.001
Triglycerides (SD)	1.14 (1.06 to 1.22); *p* < 0.001	1.18 (1.07 to 1.31); *p* = 0.002
Triglycerides / high-density lipoprotein cholesterol ratio (SD)	1.11 (1.05 to 1.17); *p* < 0.001	1.20 (1.10 to 1.32); *p* < 0.001
Lipoprotein (a) (SD)	1.07 (0.99 to 1.15); *p* = 0.079	0.96 (0.88 to 1.05); *p* = 0.341
No Hypercholesterolemia	1	1
Hypercholesterolemia	1.10 (0.93 to 1.29); *p* = 0.270	1.10 (0.89 to 1.36); *p* = 0.357
No use of lipid-lowering medication	1	1
Lipid-lowering medication	1.07 (0.87 to 1.31); *p* = 0.521	0.89 (0.67 to 1.19); *p* = 0.442
Estimated glomerular filtration rate (SD)	1.03 (0.92 to 1.14); *p* = 0.639	0.94 (0.79 to 1.11); *p* = 0.466
Normal estimated glomerular filtration rate	1	1
Estimated glomerular filtration rate < 60 mL/min/1.73 m^2^	1.19 (0.90 to 1.59); *p* = 0.225	2.06 (1.44 to 2.95); *p* < 0.001
High-sensitivity C-reactive protein (SD)	1.27 (1.18 to 1.36); *p* < 0.001	1.21 (1.10 to 1.32); *p* < 0.001
No hepatic steatosis	1	1
Hepatic steatosis	1.15 (0.98 to 1.34); *p* = 0.084	1.11 (0.90 to 1.36); *p* = 0.331
No previous non-fatal myocardial infarction	1	1
Non-fatal myocardial infarction	1.24 (0.95 to 1.62); *p* = 0.117	1.81 (1.02 to 3.23); *p* = 0.044
No previous non-fatal stroke	1	1
Non-fatal stroke	1.70 (1.20 to 2.42); *p* = 0.003	3.04 (1.71 to 5.43); *p* < 0.001

*Cox proportional hazard models adjusted for the baseline age. The following continuous variables were additionally adjusted for the use of antihypertensive medication (systolic and diastolic blood pressure), glucose-lowering medication (random glucose and glycated hemoglobin) and lipid-lowering medication (total cholesterol, low-density lipoprotein cholesterol, high-density lipoprotein cholesterol, total cholesterol / high-density lipoprotein cholesterol ratio, triglycerides, and triglycerides / high-density lipoprotein cholesterol ratio).

** SD = standard deviation: one SD corresponded to 16.4 years for age, 491 euros for household equivalent income, 18.6 g/day for alcohol consumption, 9.43 cm for body height, 15.2 kg for body weight, 4.74 kg/m2 for BMI, 13.8 cm for WC, 9.78 cm for hip circumference, 8.87% for waist-to-hip ratio, 8.11% for waist-to-height ratios, 20.9 mm Hg for systolic blood pressure, 11.2 mm Hg for diastolic blood pressure, 1.67 mmol/L for random glucose, 0.90% for glycated hemoglobin, 1.10 mmol/L for total cholesterol, 0.92 mmol/L for LDL-C, 0.40 mmol/L for HDL-C, 1.86 for total cholesterol / HDL-C ratio, 1.18 mmol/L for TG, 2.14 for triglycerides / HDL-C ratio, 286 mg/L for lipoprotein (a), 2.50 mg/L for hs-CRP, and 19.1 mL/min/1.73 m^2^ for eGFR.

In women, age (+ 614% per one SD) was the strongest risk exposure for all-cause mortality followed by previous history of non-fatal stroke. Specifically, women with previous history of non-fatal stroke had a 204% higher chance of all-cause mortality when compared with no previous non-fatal stroke. Other risk factors associated with all-cause mortality were previous history of eGFR < 60 mL/min/1.73 m2 (+ 106%), non-fatal myocardial infarction (+ 81%), lower education (+ 78%), use of glucose-lowering medication (+ 75%), being current smoker (+ 62%), type 2 diabetes (+ 62%), being sedentary (+ 42%), use of antihypertensive medication (+ 39%), being obese (+ 32%), having higher WC (+ 29% per one SD), waist-to-height-ratio (+ 24% per one SD), body weight (+ 23% per one SD), waist-to-hip-ratio (+ 21% per one SD), higher values of hs-CRP (+ 21% per one SD), hip circumference (+ 20% per one SD), total cholesterol/high density lipoprotein cholesterol (+ 20% per one SD) and triglycerides/high-density lipoprotein cholesterol ratios (+ 20% per one SD), having higher BMI (+ 18% per one SD), triglycerides (+ 18% per one SD), and glycated hemoglobin (+ 14% per one unit). Contrary to that, LDL-C (− 14% per one SD) was associated with lower all-cause mortality. On the other hand, household equivalent income, living without a partner, alcohol consumption, body height, systolic and diastolic blood pressure, random glucose, total cholesterol, LDL-C, Lp(a), eGFR, and previous history of hypertension, hypercholesterolemia, use of lipid-lowering medication, and hepatic steatosis were not associated with all-cause mortality in women.

### Prediction of combined risk factors for all-cause mortality

To identify the most important predictors for all-cause mortality, we calculated sex-specific Cox regression models which initially featured all markers showing statistical significance in the age-adjusted single regression models. On this initial model we applied a backward selection algorithm, which only kept markers with a *p* < 0.001. In men, we identified age, living without a partner, being current smoker, previous history of type 2 diabetes and high hs-CRP levels as the most important predictors for all-cause mortality (Table 3). The model including these markers revealed a strong discrimination indicated by a C-statistic of 0.854. In women, only previous history of type 2 diabetes and high hs-CRP levels were kept in the prediction model, which showed a strong discrimination indicated by a C-statistic of 0.877.

**Table 3:** Sex-specific prediction models* (hazard ratios [HR] [95% confidence interval {CI}; p-value]) for all-cause mortality under consideration of all significant markers in the single models of the associations of risk factors with all-cause mortality stratified by sex (*n* = 3,803).

Parameter	All-cause mortality HR (95% CI); *p*-value
	Men (*n* = 1,856)
Age	1.10 (1.10 to 1.11); *p* < 0.001
Living without a partner	1.78 (1.41 to 2.24); *p* < 0.001
Current smokers	1.76 (1.41 to 2.20); *p* < 0.001
Type 2 diabetes	1.83 (1.48 to 2.25); *p* < 0.001
High-sensitivity C-reactive protein	1.07 (1.03 to 1.11); *p* < 0.001
	C-statistic = 0.845
	Women (*n* = 1,947)
Type 2 diabetes	1.70 (1.28 to 2.15); *p* < 0.001
High-sensitivity C-reactive protein	1.07 (1.03 to 1.12); *p* = 0.001
	C-statistic = 0.877

* Results are derived from Cox regression under application of a backward selection with a *p* ≥ 0.001 for removal. Harrell’s C-statistic was calculated after Cox proportional hazards regression to assess model discrimination, reflecting the concordance between predicted and observed event times.

### Nomogram of combined risk factors for all-cause mortality

**Fig. 1:**
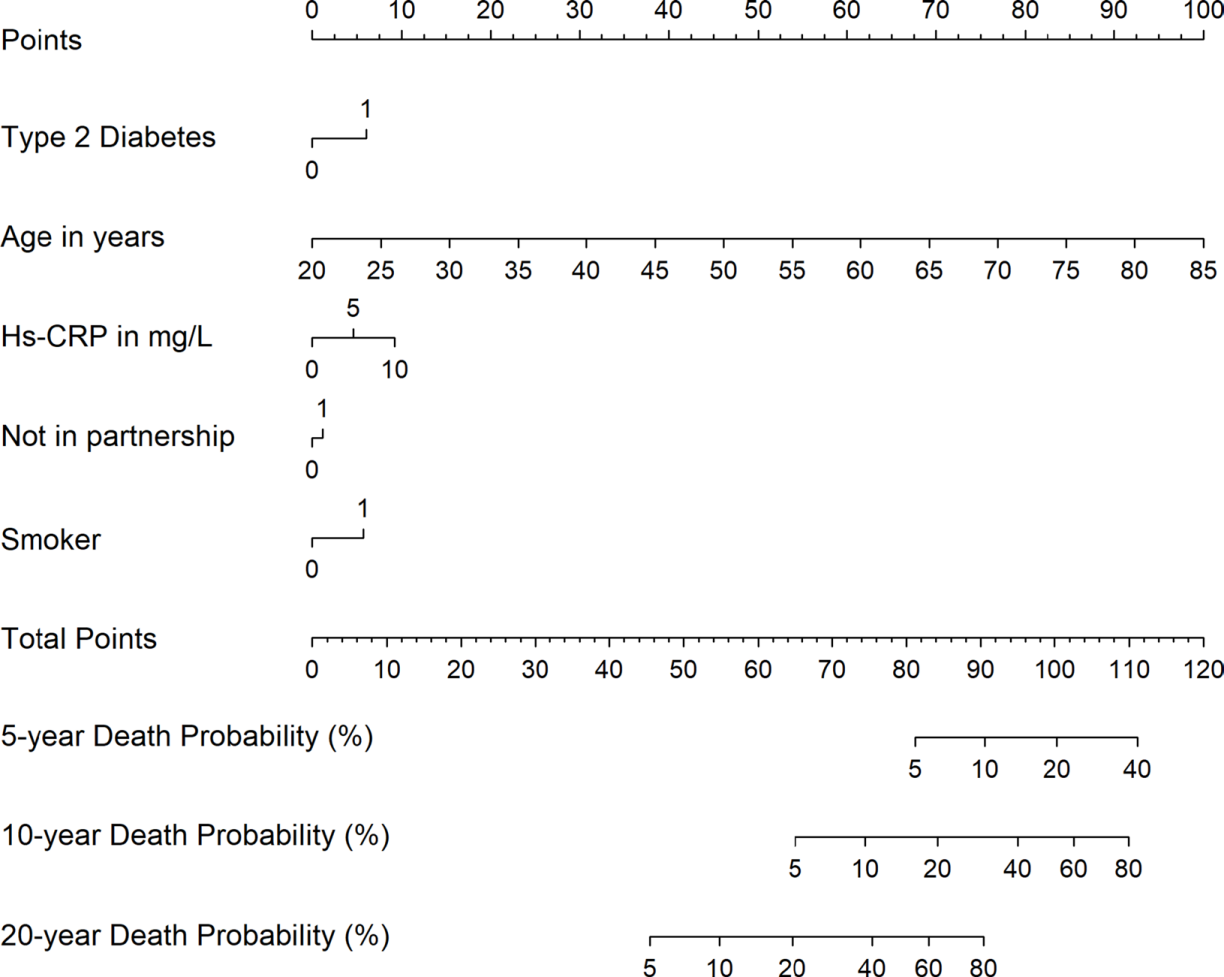

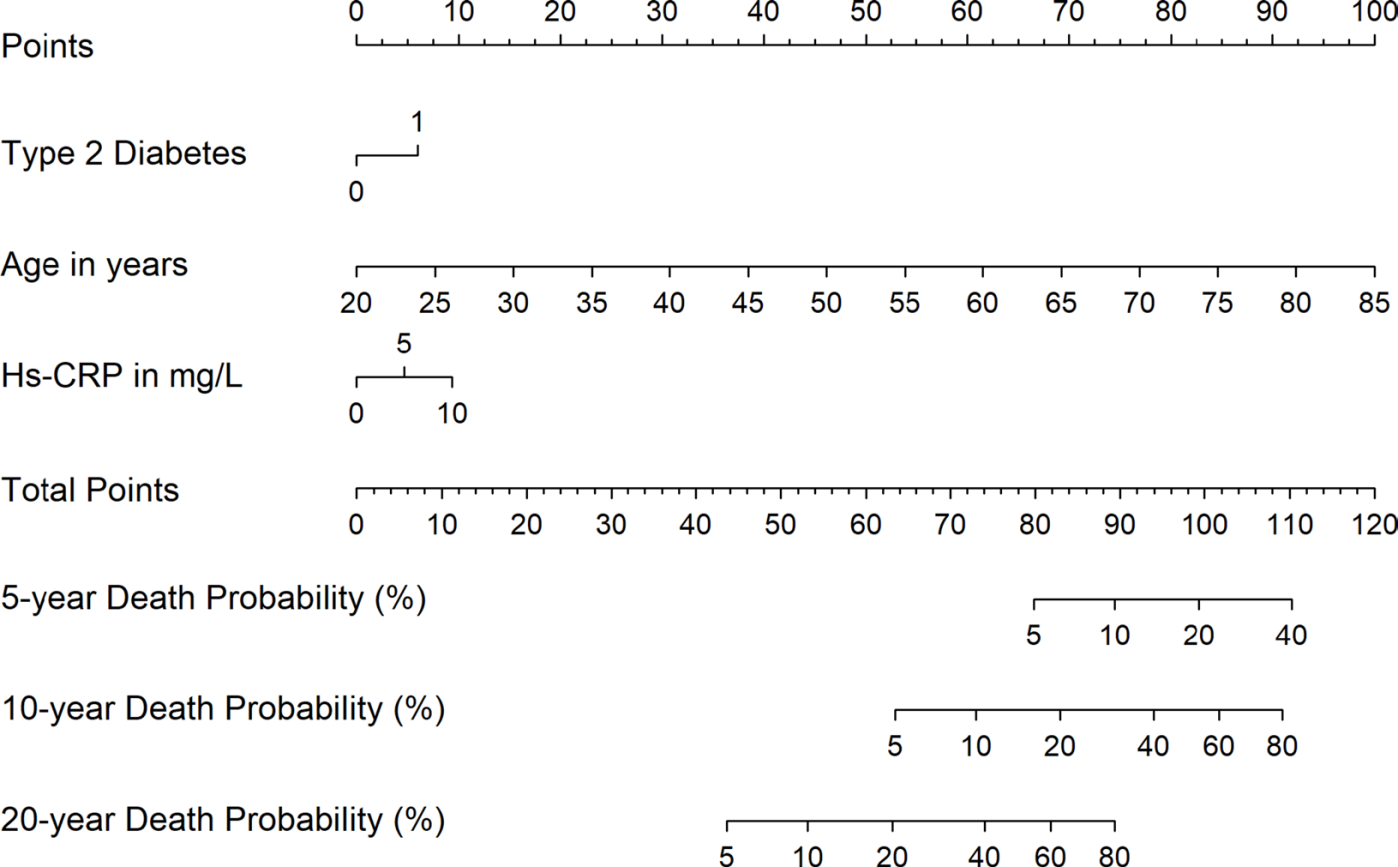
The nomogram for men (**A**) and women (**B**) for all-cause mortality risk in the Study of Health in Pomerania (SHIP). Each clinical predictor is assigned to a corresponding point value, from which a vertical line is projected to intersect the points axis above. The individual points value for each predictor were aggregated to obtain the total points score. Based on this cumulative score, vertical projections lines were drawn downwards to estimate the corresponding 5-, 10- and 20-year all-cause death probability.

In men, a total score of 100 points corresponded to a 20% probability of death within 5 years, while scores of 84 and 65 points indicated a 20% mortality risk within 10 and 20 years, respectively (Fig. 1A).

In women, a total score of 99 points corresponded to an estimated 20% probability of death within 5 years, whereas scores of 83 and 63 points were associated with 20% mortality risk within 10 and 20 years, respectively (Fig. 1B).

## Discussion

In this large prospective cohort, we identified several sex-specific predictors of all-cause mortality. For men, type 2 diabetes, living without a partner, being a current smoker, older age and elevated hs-CRP were associated with all-cause mortality. For women, we found that just type 2 diabetes and elevated hs-CRP were the most important predictors of all-cause mortality. Importantly, in both sexes, type 2 diabetes conferred the largest relative all-cause mortality risk.

### Type 2 diabetes

The single strongest risk factor in both men and women was type 2 diabetes, which is known to roughly double the risk of premature death^21^. Our finding that type 2 diabetes carries the highest relative hazard further underscores its significant impact on survival. On the other hand, while our analysis showed that men with type 2 diabetes had a 13% higher risk of all-cause mortality compared with women with type 2 diabetes (83% vs. 70%), a large systematic review and meta-analysis^22^, including 5,2 million participants, showed the opposite, that women with type 2 diabetes had 13% greater risk of all-cause mortality when compared to men. This discrepancy may be explained by the substantial heterogeneity across the studies included in this meta-analysis^22^. We did not find any novel sex-specific effect of diabetes beyond these previously reported findings, but our results reinforce the critical importance of type 2 diabetes control for survival in both sexes.

### Partnership status and mortality

We found that men living without a partner (unmarried, divorced, or widowed) had a markedly increased (+ 78%) all-cause mortality risk, whereas women’s mortality was unaffected by partnership status. This pattern echoes earlier German and global studies. The population-based MONICA/KORA cohort study^23^, including 3,596 men and 3,420 women, aged 45 to 74 years, showed that men living alone had nearly double risk (+ 96%) for all-cause death compared to married men, whereas no significant association was seen in women. Moreover, a systematic review and meta-analysis^24^ of 7.9 million individuals found that never being married conferred a substantially greater (+ 9%) all-cause mortality risk in men, when compared to never married women. The health benefits of marriage or cohabitation are well documented; marriage offers social support and can reduce unhealthy behaviors such as poor diet, smoking or heavy alcohol use^25–27^. In contrast, social isolation (often proxied by living alone) may increase stress, loneliness, and adverse physiological responses^28^. However, a recent study^29^ with 1,2 million Norwegian individuals born between 1955 and 1975, showed that while in the general population non-partnered participants presented greater mortality risks (hazard ratios of 1.59 for men and 1.47 for women), after accounting for twin effects the consequence of being in a partnership was not a significant predictor of mortality. These findings suggest that shared genetic and environmental factors might have a greater causal effect than partnership. Nevertheless, our results suggest that partnered relationships protect men’s longevity more strongly than women’s, consistent with the hypothesis that men derive larger social and behavioral benefits from marriage^30–32^.

### Smoking and lifestyle

As expected, current smoking was a key predictor of all-cause mortality in men. Cigarette smoking is a leading global cause of death, and even low-intensity smoking greatly elevates the risk. For example, in a large study realized in the United States of America^33^, using data from the National Health and Nutrition Examination Survey (NHANES) with 30,674 participants, smokers of < 20 cigarettes / day had a 54% higher risk for all-cause mortality and heavy smokers (≥ 40 cigarettes / day) nearly triple this risk when compared to non-smokers. A recent study^34^ with 1,48 million of adults from the United States of America, United Kingdom, Norway, and Canada, aged 20 to 79 years and followed for 15 years showed that current smokers had higher hazard ratios for all-cause mortality compared with never smokers (2.8 for women, 2.7 for men). Importantly, between 40 and 79 years of age, the smokers had a shorter lifetime survival (13 years for men and 12 years for women). Moreover, smoke cessation was associated with longer survival, mainly before 40 years of age. Smoking cessation of less than 3 years saved 5 years of life lost and cessation for 10 or more years saved 10 years of life lost leading to similar survival of never smokers.

These relative risks mirror our finding that smoking remained one of the most significant risk factors for all-cause mortality in men. We did not identify smoking as a significant predictor in women, likely reflecting lower prevalence or less smoking intensity in this group. Nonetheless, the harmful effects of tobacco are well-known for both sexes.

### Aging

Older age was an important all-cause mortality predictor in men. Advancing age increases the proportion of deaths due to cancer and non-cancer causes, particularly in the oldest individuals, those with frailty, and those with higher comorbidity or biological aging markers. Aging is associated with chronic low-grade inflammation (inflammaging)^35–37^, which increases the risk of mortality and age-related diseases such as diabetes^38^, cardiovascular diseases^39^, frailty^40,41^, and cancer^40,41^.

A recent study^38^ using data from the National Health and Nutrition Examination Survey (NHANES) with 41,634 participants showed that the joint presence of low-grade inflammation, characterized by elevated serum C-reactive protein, and diabetes has been associated with higher odds of biological aging acceleration and premature all-cause mortality. Frailty is a particular risk for increased overall mortality, often acting synergistically with age.

A previous study^40^ with 82,037 patients, aged 70 years or older, receiving surgical treatment for cancer showed that the cumulative incidence of all-cause death at 5 years was 56.6% for those with frailty compared to 34.9% for those without frailty. Another study^41^ with 1,291 adults, aged 60 years or older, who survived for one or more year since cancer diagnoses, identified from the 1999–2010 NHANES showed that higher allostatic load score, which incorporated measures of inflammation, metabolic homeostasis, and cardiovascular condition that reflects chronological aging, was positively associated with a 52% higher all-cause mortality when compared with lower allostatic load score.

Unexpectedly, our results showed that age was not associated with all-cause mortality in women, which may reflect a combination of methodological, social as well as hormonal factors. Statistically, age could have a non-linear effect (i.e. the hazard ratio is not constant per year of age), which may thus attenuate the observed relationship between age and mortality in our analysis. Moreover, if selection bias such as “healthy survivor” effect is present, then age itself would show a weaker link with mortality, as women who reach to advanced ages tend to be healthier. Socially, women have known advantages that can blunt the age-mortality gradient. For example, large epidemiologic studies indicate women’s mortality is often more strongly tied to social determinants (education, income, early-life conditions) whereas men’s mortality is driven more by behavioral factors^42^. Women who reach older ages frequently have had better early-life environments – one study found that later menopause (a marker of slower aging) is associated with better childhood nutrition, lower stress and higher socioeconomic status, all of which contribute to longevity^43^. Hormonal differences may also play a key role as premenopausal women have much lower rates of cardiovascular disease than men of the same age, a difference attributed to estrogen’s protective effects on the heart and vasculature^44^. This means women effectively enjoy a delayed onset of major causes of death, narrowing the age-mortality gap until after menopause. Even postmenopausal factors may help explain our findings as timely use of menopausal hormone therapy has been linked to reduced all-cause mortality in younger postmenopausal women and could further obscure the age effect^45^. In sum, these statistical nuances, coupled with women’s genetic/early-life advantages, healthier behaviors, and hormonal protection, may collectively explain why we did not observe a significant effect of age in mortality risk in our sample of women.

### Inflammatory risk factors

After sociodemographic factors, inflammation, characterized by elevated hs-CRP levels, was the most significant risk factor for all-cause mortality in both men and women. Elevated hs-CRP was a robust predictor in both sexes, reflecting the role of chronic inflammation in mortality. This is consistent with pooled evidence from a previous meta-analysis^46^ with 83,995 participants from 14 studies that found that subjects in the highest hs-CRP category had a 75% higher risk for all-cause mortality than those in the lowest. Another study^47^ with 14,238 participants, aged 35 to 74 years, from the Brazilian Longitudinal Study of Adult Health (ELSA- Brazil) found that individuals in the fourth quartile of hs-CRP had a 95% higher risk of all-cause mortality when compared to participants in the first quartile. Finally, a recent study^48^ with 5,294 stable patients with hypertension, up to 20 years of follow-up, from the AngloScandinavian Cardiac Outcomes Trial (ASCOT) showed that compared to the lowest tertile of hs-CRP levels, individuals in the highest tertile had a 25% higher risk of all-cause mortality.

Metabolic and inflammatory changes, including mitochondrial dysfunction and oxidative stress, are linked to worsening health and higher mortality in older adults^49^. Hence, inflammation acts both as a marker and a driver of the decline in resilience and the rise in chronic diseases and death with age^35–37^.

### Nomogram model for all-cause mortality

The development of death risk stratification models allows the identification of these risks and possible prevention or treatment of them aiming to delay the progression of death probability. Moreover, these models also aim to lessen unnecessary interventions in individuals at low risk (rationalizing the provision of healthcare resources).

Noteworthy, as we did in our analysis, risk models for all-cause mortality usually include cardiovascular risk factors, because they are also strong predictors of death from any cause, not just cardiovascular deaths^50,51^. Commonly used models include traditional cardiovascular risk factors such as age, smoking, blood pressure, type 2 diabetes, body mass index, and cholesterol levels. In our models we have included type 2 diabetes, age, hs-CRP partnership and smoking for men and type 2 diabetes, age and hs-CRP for women, all of them may be considered predictors of cardiovascular death. We decided to include the nomogram in our analysis to allow a better visualization of our findings.

### Strengths and limitations

Key strengths of our study include the population-based design, large sample size, and long-term follow-up of the SHIP-START-0 cohort. We analyzed men and women separately, allowing detection of sex-specific associations. Risk factors were comprehensively assessed (socioeconomic, lifestyle, anthropometric, clinical) and our models were adjusted for medication use. Some limitations should be noted. First, we excluded 505 participants (12% of the total sample) because of missing values data for any of the variables considered which might have introduced some bias in our analyses. Second, unmeasured confounding may influence associations. For example, men living alone may differ in unmeasured ways (e.g. social support) from those with partners. Third, physical activity and some covariates were self-reported, potentially causing misclassification. Fourth, our cohort is from Northeast Germany, which may limit generalizability to other populations. Fifth, we focused on all-cause mortality, so we cannot attribute findings to specific causes (e.g. cardiovascular vs. cancer). Finally, our analysis included a large number of variables. The selection process of these variables may lead to collinearity and overfitting, usually as a result of the multidimensionality and complexity of the clinical data^52^. Nonetheless, the consistency of our results with prior studies supports their validity.

## Conclusions

Our sex-stratified analysis in a Northeast German population confirms that social and lifestyle factors influence mortality differently in men and women. Notably, well-established risk factors such as type 2 diabetes and inflammation were associated with mortality in both sexes, while being without a partner, current smoking, and older age were associated with mortality only in men. These findings underline the importance of tailored prevention strategies that address both biological and social determinants of health. Optimizing type 2 diabetes management remains critical for improving survival in both sexes, whereas reducing social isolation could be particularly advantageous for men. Future studies should investigate the mechanisms underlying these sex differences and assess targeted preventive strategies.

## Data Availability

The data of the SHIP study cannot be made publicly available due to the informed consent of the study participants, but it can be accessed through a data application form available for researchers who meet the criteria for access to confidential data at [https://fvcm.med.uni-greifswald.de](https:/fvcm.med.uni-greifswald.de).
